# Involving clients and their relatives and friends in psychiatric care: Case managers' experiences of training in resource group assertive community treatment

**DOI:** 10.1002/pchj.1

**Published:** 2012-06-10

**Authors:** Tommy Nordén, Anders Eriksson, Anette Kjellgren, Torsten Norlander

**Affiliations:** 1 Evidens Research and Development CenterGöteborg, Sweden; 2 Department of Psychology, Karlstad UniversityKarlstad, Sweden

**Keywords:** ACT, case manager, optimal treatment, RACT, resource group

## Abstract

The purpose of this project was to do a qualitative study of an integrated and flexible ACT model, the Resource Group Assertive Community Treatment (RACT), as seen from the perspective of case managers in training. The resource group normally consists of the client, the case manager and other available personnel in the medical and support areas, as well as family members. Nineteen theses were randomly chosen from a set of 80 theses written by a group of Swedish trainee case managers. The exams were conducted as case studies and concerned 19 clients with psychotic problems, 11 men and 8 women. “The Empirical Phenomenological Psychological Method” was used in the analysis, which generated five overarching themes: (a) the RACT program; (b) the resource group; (c) the empowerment of the client; (d) progress in treatment; and (e) the case manager. These together constituted a “therapeutic circle,” in which methods and tools used within the RACT made it possible for the resource group to empower the clients who, as a result, experienced progress with treatment, during which the case manager was the unifying and connecting link.

Assertive Community Treatment (ACT) is a community-based treatment and rehabilitation program developed during the 1970s and 1980s that was intended primarily for individuals with long-term illness, who, as a group, were large-scale consumers of the resources available within psychiatric in-patient care (Stein, 1990; Stein & Santos, 1998; Test & Stein, 1978). An ACT team is a multidisciplinary team that works with intensive clinical case management focused first and foremost on treatment. The treatment is carried out in the patient's own immediate surroundings rather than at a clinic or other health-care setting. There are at present a number of different ACT models and new experience and research results are reported constantly, all leading to further development (van Veldhuizen, 2007). The original variant that was dominant in the USA during the 1970s and 1980s was carried out by a purely psychiatric health-care team, in which all members of the team were capable of working with all of the clients and where all team members could have a case management function (Lewin Group, 2000). In any such team there was at least one full-time psychiatrist, two full-time nurses, and also staff with specialized knowledge of substance abuse and/or with experience with work rehabilitation. A total of 10–12 professional team members together had responsibility for approximately 100 clients. This primary variant of ACT is referred to here as the orthodox ACT model, as distinct from the more modern integrative ACT models.

The orthodox models, as used in the USA, were able to produce satisfactory evidence-based interventions (Bond, Drake, Muesser, & Latimer, 2001), but further discussion of the effectiveness of these models is needed. The orthodox ACT models have been only slightly modified during the past 30 years (Bond & Drake, 2007) even though the health-care system and many different social factors have changed dramatically. Comparative studies of ACT in Western Europe do not report results comparable with the good results from the USA (e.g. Burns, Fioritti, Holloway, Malm, & Rössler, 2001; Fiander, Burns, McHugo, & Drake, [Bibr b13]). These differences might be a result of the differences between the standard care given to the control groups in Europe and those in the USA. In Western Europe standard care is based on the social-psychiatric paradigm, in contrast with the care given in the USA, where standard care may be associated with longer admissions (Bak et al., 2007). This has resulted in the development of ACT with so-called Assertive Outreach in the UK, which provides both treatment and social support through the cooperative efforts of personnel from psychiatry and social services (Malm, 2002). It is difficult to adapt the orthodox models requiring large teams operating 24 hr per day to rural settings where problems arise in providing continuity and where long travel distances are common (Burns et al., 2001). Along with the fact that there is a steady increase in knowledge about efforts involving early intervention in connection with psychological disorders (Killackey & Jackson, 2007) reconceptualizing the target population for ACT is becoming ever more important, as Bond and Drake (2007) observe. Perhaps the service no longer should be aimed only at the most severely mentally ill clients, but might also be more flexibly used for a broader range of clients.

The integrative ACT models are characterized by greater flexibility and closer proximity to the clients (Malm, 2002). More recent research (e.g. Thornicroft & Tansella, 2009) emphasizes the importance of personal contact with a specially qualified case manager and a relationship in which the concept of “shared decision making” is central. The goal of shared decision making is to formalize the participation of the clients in contributing to their own care. An example of a modern integrative ACT model is one developed in Holland, known as Function-ACT (or FACT; van Veldhuizen, 2007). In this version of ACT (also called “Flexible” ACT), the team takes care of both those clients who have intense needs for treatment and also those whose needs for treatment are less intense (Bak et al., 2007) and every client has an individual case manager. Continuity during treatment is ensured by making it possible for clients who have previously had serious problems to continue to remain in treatment during periods when they are feeling better and don't need as intensive care. Inasmuch as clients find themselves in different psychological states with respect to the need for interventions, the case loads for case managers are distinctly higher than the case load of approximately 10 that constitutes the normal load within orthodox ACT. Case-loads of approximately 15–25 clients per case manager are the normal within FACT. Assertive outreach is put into practice when case workers make both announced and unannounced visits to the homes of clients and where these case workers strive to teach the clients effective coping strategies that are adapted to the special needs and health status of the clients. Intensive treatment and support at an ACT level is provided due to the high risk of admission because of early signs of relapse. Symptomatic remission criteria have been developed for schizophrenia (Harvey et al., 2003; Helldin, Kane, Hjärthag, & Norlander, 2009) and research results indicate (Bak et al., 2007) that severely mentally ill clients who are treated according to FACT move more quickly towards remission than clients who are treated by following the standard care treatment approach.

Even if FACT is more flexible than orthodox ACT in many ways and is better adapted to the needs of the client, it is the tradition that originated in the work of the New Zealander Ian Falloon (1999) that has most radically taken hold in the field of shared decision making and the empowerment of clients. The idea was that care givers would collect knowledge about the best evidence-based methods that are used within psychiatry and apply these in the treatment system, “Optimal Treatment” (Malm & Lindgren, 2002). The evidence based treatment strategies used in Optimal Treatment (Falloon et al., 2004) are numerous: (a) Minimally effective antipsychotic drug strategies targeted at changing symptom profiles; (b) education of clients and informal care givers using stress management strategies; (c) assertive case management; (d) goal-oriented social and occupational skills training and supported employment; and (e) specific pharmacological and/or psychological strategies for residual or emerging symptoms. The method was developed through an international research project called the Optimal Treatment Project (Falloon, 1999), which is an ongoing project begun in 1994. The treatment program as a whole has been scientifically researched and field-tested in a number of countries, each of which has its own particular system for providing health care and welfare support (e.g. Lurigio, Fallon, & Dincin, 2000; Malm, Ivarsson, Allebeck, & Falloon, 2003; Mastroeni et al., 2005; Mizuno et al., 2005). The Optimal Treatment model is built around the basic theme that the subjects themselves set the goals for their treatment and have a decisive impact on how the treatment is to be designed (Nordén, Ivarsson, Malm, & Norlander, 2011). This model has been given a number of different names in addition to Optimal Treatment, among them Integrated Care, Integrated Psychiatry, Integrated Mental Health Care, but since the model continues to be developed with ever greater emphasis on the central position of the client through the participation of a so-called resource group, we choose from now on to call the model the “Resource group ACT” or RACT.

RACT is distinctive in that the ACT team consists not only of professionals, but also of the client and his/her significant others. The philosophical position is that if all activity is to originate in the needs and wishes of the client then the client cannot be excluded, as was the case with the orthodox ACT team. To do so would be to preserve the older pattern in which caregivers within psychiatry and social services go over the heads of the clients to make the decisions, thus preserving the belief that the care giver knows better than the client what the needs of the client are. By the early 2000s, the new form of the ACT team had come to be called the Resource Group (Malm, 2002). The existence of the Resource Group, that is the RACT team, is not opposed to professional team meetings or conferences in order to discuss interventions for the most seriously ill patients, nor is the team opposed to case managers having a supporting network among themselves. However, even if such meetings can take place, the ultimate goal is to then see to it that all interventions take place as soon as possible in the form of assertive outreach service by the RACT team and in close and respectful cooperation with the client. The Resource Group probably has special importance in the Swedish version of RACT because responsibility for Swedish psychiatric care is shared by two government units, one the regional County Council (*Landstinget*) with responsibility for administering health care, the other the local municipal government (*Kommun*) with responsibility for administering social care and interventions (Sjöstedt, 2007). As a consequence there is a significant risk that the client will, so to speak, fall between two chairs, that is between the two government agencies that share responsibility. Through the auspices of the RACT team, personnel from these two agencies should be able to communicate with each other more easily. An additional advantage provided by the RACT team's flexibility, as compared with the traditional ACT team, is that a variety of specialists, such as personnel from the Swedish National Employment Agency (*Arbetsförmedling*) and the Swedish National Prison and Probation Administration (*Kriminalvårdstyrelsen*), may be added to the Resource Group when their competence is needed.

During a 3-year period, 80 people took part (18 men and 62 women) in a nationwide training program in Resource group ACT (RACT) at five different locations in Sweden. The participants were primarily nurses and social workers employed in social welfare care or psychiatry who would for the first time have access to further training in case management (CM) and modern integrative Assertive Community Treatment. During the final year, each was given the opportunity to work with a client while employing the RACT program under the guidance of a supervisor familiar with the integrated care approach. As their final assignment they were required to complete a case study of a client whom they had treated with the help of RACT.

The aim of the current investigation was to qualitatively study the Resource group Assertive Community Treatment (RACT) from the perspective of the trainee case managers in order to expand our knowledge of these individuals' views on RACT after 3 years of training in the use of the method.

## Method

### Participating case managers and clients in the training program

On the basis of the case studies that the case managers (CM) had prepared as the final exam, it became possible to summarize some quantitative findings concerning the clients. It became clear that, of the 80 clients described (39 men and 41 women) approximately 65% had diagnoses that lay within the psychotic spectrum while the other clients were found to display the whole range of typical psychiatric diagnoses, such as delusional syndrome, addiction syndrome, depression, panic disorder, anxiety disorders, obsessive-compulsive disorder, behavioral disorders, personality disorders, and attention disorders. In addition, it became evident that 69% were single and 22% were divorced. The mean age of the participants was 40.28 years (*SD* = 11.89 years). Most of them were unemployed or were receiving compensation while on sick leave; only 4% had a full-time job, but 77% had their own apartment. Except for the CM and the physician the client got to personally choose who was to be part of the RACT team. In all of the 80 Resource Groups, the clients had nominated professionals in whom they had particular confidence and, in most of the resource groups (73.8%) even relatives were included. On average the Resource Group had five meetings over the year, during which the CM-in-training worked together with his or her client. In addition to these meetings, the CM and the client were in close and repeated contact with each other. In the RACT method, the clients are supposed to formulate the goals they wish to reach on their way to reaching a state of better psychological well-being. In connection with the final phase of the training program, the trainee CMs wrote their final exams in the form of case studies in which they described the activity over the preceding years. An overall review showed that two of the 80 participants had become worse and for 12 there apparently was no change, but 45 (56.3%) clients were judged to have shown some kind of improvement and 21 (26.3%) to have shown “major improvement.” In the evaluation of the importance of the Resource Group in achieving progress it was found (using Pearson's *r*) that the greater the number of Resource Group meetings, the greater the progress for the client (*r* = 0.48, *p* = .018).

### Ethical considerations

All of the 80 clients received information orally and in writing about the conditions for participation in the training project. It was explained that each CM-in-training would submit a final paper in which a case study would be presented. Each paper would be published and discussed in a seminar. The clients were informed that these papers would be designed so that the client's anonymity would be maintained. In addition, each client was informed of his or her right to break off participation in the training project at any time without needing to give a reason. A contract was drawn up and was signed by both the client and the trainee CM. Given these conditions, the Swedish rules on ethics allow those responsible for a training program to compile student reports to create an article as a contribution to the evaluation and follow up of such a program.

### Participants in the qualitative study

From the total of 80 case studies, we randomly selected 20 project reports on clients who suffered from psychosis. The reason for selecting those with a diagnosis of psychosis as a study group was that this group is a large group in need of care and coordinated support, and it is also a group that is easily distinguished from the total population so that it is easier to work with them and isolate the results. One of the case studies was excluded because it became apparent on close examination that this client did not suffer from a problem related to psychosis. The 19 case studies of clients with psychosis that were included pertained to 11 men and 8 women.

### Processing the data

The Empirical Phenomenological Psychological Method (EPP method) devised by Gunnar Karlsson (1995) was used in processing the data. The method consists of several different steps and within these steps the text is to be subdivided into small content-bearing units referred to as “meaning units” (MU). This procedure followed no grammatical rules. Instead, the subdivision was made where the content and meaning of the text changed. In order to make an analysis of the MUs possible when they were to be dealt with outside the original context, each MU was transformed so that the psychological and contextual implications were stressed. The analysis yielded 1,076 transformed MUs that in turn generated 39 categories. Each category illustrated a special perspective of the phenomena studied, and each category was described in a synopsis. To control for the reliability of the results of the study, the Norlander Credibility Test (NCT), designed for phenomenological analysis, was used (Åsenlöf, Olsson, Bood, & Norlander, 2007; Bergman & Norlander, 2005; Edebol, Bood, & Norlander, 2008; Janson, Archer, & Norlander, 2005; Niklasson, Niklasson, & Norlander, 2010). Ten synopses were randomly chosen and then five transformed MUs were randomly chosen from each of the 10 categories. Two assessors then had the task of independently assigning the 50 transformed MUs to the synopses that they believed to be most satisfactory. The assessors' average result was 80%, which is in line with previously published results (Åsenlöf et al., 2007; Bergman & Norlander, 2005; Edebol et al., 2008; Janson et al., 2005; Niklasson et al., 2010). Finally, the 39 categories were easily related and subordinated into seven all-embracing categories, so called index categories. Where examples of MUs are given in this text, it is the original raw MUs that are presented in quotation marks below. Table 1 provides a summary of the index categories as well as the number of MUs in each category.

**Table 1:** Overview: Index Categories, Categories and Number of Content Bearing Units (Meaning Units (MU))

Index categories	Categories	No. of MU
**1 Client**	1 Client's situation within RACT	38
	2 Client's psychosis-related problems before RACT	50
	3 Client's other difficulties before RACT	30
	4 Client's problems with relationships	15
	5 Client's wishes concerning the development plan	69
	6 Setbacks experienced by client	14
	7 Client's successes within RACT	64
**2 CM**	8 CM's work within RACT	21
	9 CM's daily achievements	56
	10 CM's experiences of differences between RACT and previous approaches	37
	11 Difficulties experienced by the CM during work	22
**3 Resource Group**	12 Participants in the main Resource Group	39
	13 Resource Group activities	67
	14 Practical advices for carrying out the meetings of the Resource Group	9
	15 The Small Resource Group	41
**4 Medical care**	16 Hospital Admission	10
	17 Somatic health and hospital care	30
	18 Dental care	10
	19 Physician's functioning within RACT	10
	20 Problems with accessibility to physicians	11
	21 Side effects of medicines	10
	22 Problems with finding the correct dose of medicine	11
	23 Problems with medication	29
**5 Community efforts**	24 Cooperation in community efforts	31
	25 Residential support	34
	26 Monetary support for activities	31
	27 Economic issues and the trustee	18
**6 Relatives**	28 The relatives' situation before the RACT program	29
	29 The relatives' wishes and attitudes toward RACT	19
	30 The relatives' situation after the RACT program	16
	31 Social network after the RACT program	29
**7 RACT program**	32 How the client has experienced the RACT-materials	12
	33 How the CM has experienced the RACT-materials	28
	34 Reflections related to the RACT-literature	16
	35 Belief in the program	26
	36 Creation of alliances	28
	37 Educational efforts	29
	38 Problem solving	21
	39 Crisis plan for the client	16

*Note.* CM = case manager; RACT = Resource Group Assertive Community Treatment.

## Results and discussion

As already indicated, seven index categories emerged, each one of which consisted of several sub-categories: (a) the client; (b) the case manager; (c) the resource group; (d) medical care; (e) societal contributions; (f) relatives; and (g) the RACT program. We will first discuss each index category separately and then investigate whether or not it is possible to identify recurring themes that cast light on the essence of the phenomenological analysis that was carried out.

### The client

This index category describes the client's situation and difficulties faced before, during and directly after training in RACT. The clients frequently suffer from problems such as a lack of activities, depression, substance abuse, problems with the administration of medicine, difficulties with executive functions, and social uncertainty. The development plan that was developed together with the client was formulated by setting both short-term and long-term goals, and some of the problems named above often came up during the planning of these goals. The problems were often interwoven with each other, as may be illustrated by the following quotation from a CM: “Yet again, it is the drabness and lack of company that he wants to relieve with tranquilizers. His argument is that in the past he had so much more, now he only has half.”

Clients found it meaningful that the CM asked about their own goals and needs. An essential element of the program is that the clients formulate goals that can then be broken down into sub-goals in order to make it easier for the goals to be realized. The most common overriding goals that the clients wanted to reach were a socially richer life (“greater social interaction,” “want to become less of a loner,” “to come out into society”), better health (“to eat enough,” “to lose weight,” “to reduce stress”) and better management of daily life (“better sleeping habits,” “be able to take care of my home myself,” “take my medications myself”). In view of the fact that dealing with goal setting was carried out during training, the setbacks were relatively few compared with what were experienced as successes as measured by the number of MUs (14 setbacks compared with 64 successes). The successes were clearest as concerned the social contacts and the ability to take initiative. The level of functioning was raised and self-sufficiency strengthened as a result of the client learning more about him- or herself and about his or her illness or condition. The clearer structure that the Resource Group made possible and the improvement in taking medicines both contributed to positive developments. The level of knowledge among the participants was raised. The number of hospitalizations was reduced and the need for treatment was lowered. All 19 of the clients who were studied experienced improvement in at least one respect, and of particular importance was the improvement in quality of life resulting from the use of RACT: “The client has become more conscious of her problems and she knows what she needs to do in order to become better.” The successes in the sub-group were also in keeping with what could be reported for the entire study group of 80 participants, among whom it was evident that approximately 83% were judged to have made at least moderate progress and in some cases even major progress (see Methods section). As in all therapeutic treatment situations, the successes did not take place in a straight line, but were separated by repeated relapses or by reaching a plateau (Edebol, Kjellgren, Bood, & Norlander, 2009; Kjellgren, Buhrkall, & Norlander, 2010, 2011). The clients now discovered that with the help of the program and the CM it was easier to deal with temporary setbacks.

### Case manager

The CM has a key role in RACT and bears responsibility for seeing to it that all that is required in RACT is carried out as intended. The participating CMs have previous backgrounds as employees in some element of the overall health-care system. The role that the CM now took on required the CM to function in the beginning as both a helping hand for the client and to some extent as therapist. Once the program was underway, the CM became the driving force who, for example, arranged the Resource Group meetings and saw to it that everything else around the client functioned. It became apparent that what then followed was a transformation from focusing in the beginning only on care of the client to gradually transferring ever more personal responsibility to the client, which the following quotation illustrates: “To develop into a CM is basically a process entailing the appearance of insights that reveal themselves step by step. It is the client who is to be given influence and it is my task to give the client support and the tools to be able to take power again.”

The CM had to deal with questions concerning medication in addition to dealing with acute problems that could arise for the client. Making contact with family members was quite different in the RACT program than in previous programs, because in RACT the contacts were better structured and more detailed than in any of the previous medical-care situations in which the trainee CMs had been involved. The Work Book, which the CM is supposed to consult, frequently specified various techniques that are also used in cognitive behavioral therapy (CBT). RACT was also seen as being more effective than standard care in motivating those involved and the participants used the supporting documents and information in a more professional manner: “On two occasions I have been in meetings with the entire family and Social Services personnel concerning the placement of the daughter with the child's maternal grandmother.” The difficulties that the CM encountered were of two kinds, one the client's functional problems and the other the different views held by other people on how the client should be managed: “A sociogram had to be done twice. The first time he (the client) was affected by psychotic incidents, and the second time there were no results at all.” What was called for in such a situation was that the CM took on the new role as planner and leader in a completely different way than the CM had done in previous professional settings. The improved follow-up procedures and a mode of operation that gave the client a real lift in quality of life resulted in making the role of the CM a role that was experienced as positive and leading to development: “It takes a bit of courage to realize that it is I in my role as CM who must coordinate everything and maintain a certain structure while at the same time working with the client to set priorities concerning which questions are to be given the highest priority.”

### The resource group

A ground-breaking element that RACT inserts into both psychiatry and social services is a Resource Group that normally consists of the client, the CM and other available personnel in the medical and support areas, as well as family members. The total commitment of a physician in the Resource Group is seen in RACT as being of the greatest importance, something that has been found to be difficult to realize because of the severe shortage of physicians within the psychiatric care system in Sweden. The Resource Group makes decisions together and takes responsibility for seeing to it that the development plan is followed (Malm & Falloon, 2008). The client chooses the participants from her social network. The CM and the client always took part and an effort was made to include a physician; in addition to these people one or more family members and a facilitator were usually present: “The client chose those who would be able to contribute something to her recovery. She questioned her parents, the psychologist, the physician and her residential support person from the county.” The participants recommended by the client were contacted by the CM for an interview and received an inquiry concerning possible participation. Most of them agreed to participate because they saw the advantages of bringing together all available resources in one place. When it was not possible to create a Resource Group in the intended form it was found that the reason for this was that the physician was not able to make a full commitment because of demands on his or her time, or because the client's social problems made it impossible to have a meeting with several people present. The number of people who participated in the resource group in addition to the CM and the client varied from two to eight in the 19 cases.

Activities at the meetings followed the agenda that the CM together with the client had agreed on. An attempt was made to get the client, with the support of the CM, to function as the leader of the meetings if at all possible, as a fundamental position in RACT is to empower the client, to restore his or her self-confidence. In the beginning the focus was placed on setting up the group and in providing instruction concerning the client's impairment and questions about medications. The client presented his or her development plan, followed by problem-solving approaches needed to reach his goals. Plans to deal with crises by recognizing the early warning signs that indicate an imminent psychotic event were established in order to prevent new hospitalizations. These activities resulted in a strengthening of the participants' positive views by seeing to it that all successes were pointed out and everyone had a chance to express themselves. The participants understood clearly the tasks to be dealt with and thus saw clearly how responsibility was to be assigned: “The meeting becomes effective as a result of everyone being present and seeing to it that tasks and responsibilities are divided effectively among the participants.”

In order to achieve the best results, preparation in which the client actively took part in the planning and was involved as one key decision maker was essential. Unease about the meetings could be reduced by having people well known to the client take part, and this in turn made it easier to arrange get-togethers at the home of the client. “A hindrance that has occurred is that the client cancels a planned meeting at the last minute. We found the solution by meeting at the home of the client instead of at the clinic.” At the same time, some problems could be solved in direct connection with the meetings: “I took a sketchpad and easel with me on which I could write the day's agenda and then we could begin by taking up other questions not formally on the agenda as well.” The Resource Group's meetings and activities build a network around the client. During the project it became evident that the number of contact people around the client is central if progress is to be made, and also that the Resource Group plays an important role in providing instruction and various kinds of support for the relatives, something that is of particular importance in the care of schizophrenic patients (Dixon et al., 2001; Lauriello, Bustillo, & Keith, 1999). As has previously been reported, the number of Resource Group meetings is of great importance and even the size of the Resource Group appears to be essential if progress is to be made. The somewhat lesser progress seen in the groups with fewer participants that we believed we observed in our data presumably mirrors the absence of important components in successful RACT, components such as, for example, access to a physician and the full engagement of family members. The collective relational network around the client becomes smaller, which results in decreasing the number of successes. This also illustrates the importance of coordination among all the helping authorities around the client. The Resource Group also has a kind of working committee consisting of the client, someone from the social network and the CM and this “committee” met more often than the Resource Group proper, often once per week or every second week. This is called the “small Resource Group” and it takes care of more routine needs in the form of teaching, conflict resolution, planning of Resource Group meetings, and evaluation and sometimes has an occasional guest who is invited in to deal with some kind of acute matter.

### Medical care

In this index category all the activities that concern medical care in some way are included. Psychiatric problems often led to secondary somatic problems such as obesity followed by diabetes, high blood pressure, and dental problems. Even problems with the administration of medicine were frequently encountered. Clients have often been hospitalized many times in the psychiatric ward. During their year with RACT, 5 of the 19 clients experienced a decreased frequency of hospitalization and the remaining ceased having to be hospitalized. Before RACT, admission to and discharge from the hospital were the times when someone at the hospital contacted relatives and the medication plan was reviewed at the time of discharge. RACT has contributed to seeing to it that questions about medication and maintenance of contact with family members are dealt with in a more professional and structured manner. Contact with physicians via the Resource Group and follow up by the CMs together with family members has improved control of medication. Corrective measures concerned with medication have been taken in 12 of the 19 cases. These have most often been in connection with the experience that the client has not managed to take the medicine that has been prescribed, but corrective measures have also been taken in connection with changes in the type of medicine or the dosage for the prescribed medicine. An example of an intervention concerning the administration of medications might be that someone at a Resource Group meeting designated individuals to take responsibility for the following actions: (a) that someone would remind the client that the medicine would be taken at a specific time, for example in connection with a home visit; (b) that someone gave the medicine in a special dose dispenser; and (c) that someone counted the number of unopened medicine packages. As the following quotation indicates, the CM has a key role in this work: “At present, we have solved the problem of accessibility to medication by seeing to it that the medicine is kept in the residential support person's area; if the client is not at home or does not open the door during the morning visit, the day's medicines are left in dose packages in a sealed envelope. I have provided the envelopes, which are decorated with small uplifting comments. These simple actions have been much appreciated by the clients. Adherence to the schedule has been good.”

On occasion it could also be necessary to adjust the dose to match the improvements taking place in the client's condition or when something happened that triggered the crisis plan, with the result that the dose had to be increased temporarily. The side effects of the antipsychotic drugs constituted another problem that was taken up in discussion. The most common effects were weight gain and apathy, which led to offering extra training concerning diet and exercise for users in connection with a medical checkup. It was of considerable importance that the physician could participate fully in the Resource Groups meeting so that the medication plan could be modified quickly and the necessary referrals written. In the 19 cases there were three physicians who were called in when needed, six who sometimes could not come because they had other responsibilities and one where no physician could be present. Physicians were able to participate fully in only nine of the cases. In addition it could happen that a physician was replaced by another because they were employed following the so-called physician relay system. Functional impairment among the clients also led to problems with their teeth, so the CM has even helped clients to receive dental care in a way that was not previously available. In summary, the medical preventive measures have led to clear improvements in the lives of many of the clients that would not have occurred in the absence of the CMs and the activity of the Resource Group.

### Community efforts

Interactions between representatives for the county and the County Health Council have occurred in the past, but these have not been as well structured or as clear as they now are in RACT. The need for such interaction is often obvious and attitudes toward RACT usually become more positive when representatives discover that, under RACT, the load placed on these representatives is reduced. In order to facilitate implementation, what is needed are clear statements from the representatives as well as support within the organizations. A CM from each county government and from each County Health Council have occasionally been set up to make use of old contacts among employees at both: “Everyone thinks that the creation of the ACT-team (i.e. the Resource Group) has meant that ‘our clients cause fewer problems’ for the three organizations (County Health Council, Social Services, Social Psychiatry) since they get adequate help when it is needed. It also appears that the various organizations display steadily increasing confidence in the judgments of the ACT Team.” The CM and the Resource Group also cooperated in supporting several other situations. Residential support people helped the client with purchases, cleaning, laundry and other tasks of daily life, following the plan that had been laid out. It was essential to make the residential support person part of the activity because if this was not done “problems (could) arise if the residential support person did not understand the nature of a client's disability but instead believed that all that was needed was for the client to ‘shape up’ in order to be able to, for example, manage to take care of cleaning the apartment.” Many clients who are dysfunctional in some respect can be dangerously inactive if they do not receive support for becoming active and it is therefore important to coordinate efforts together with providing services such as escorts and county vocational rehabilitation. These activities were seen by the client as being worthwhile and participation decreased the risks of substance abuse or gaining weight. The clients' sub-goals could sometimes be reached thanks to taking part in these activities: “We mapped the client's day with the help of a questionnaire ‘An ordinary day.’ It became obvious that on those days on which the client did not have any activities outside her apartment, she was completely passive” and “In the new developmental plan it is explicitly stated that the client must visit the activity center at least three times each week and do so with the support of the residential support person.” Users sometimes faced difficulty in not being able to manage their personal economy. Disabilities and in some cases being financially exploited by others have led to economic problems. A trustee or financial custodian was occasionally provided to help the clients with their economy. Family members were sometimes a resource in dealing with financial matters.

### Relatives

Much more attention is given to family members in RACT than in other forms of care by making it possible for family members to be part of the Resource Group and by the CMs greater commitment to them as dictated by the program's well-structured mode of operation. The clients were given a chance to make a map of their social network and the resulting maps varied considerably in size and function. Good networks did exist, but others had dysfunctional or almost invisible networks and the need to train some clients and those closest to them to communicate became evident. In families weighed down by problems in dealing with affect, so called expressed emotion (EE), the frequency of relapse was also greater than in the more stable families, which is in line with previous research (Leff, 1996). Education about the illness and its treatment was therefore of extra importance in order to reduce the level of concern and the feelings of shame experienced by the client's family, thereby reducing EE. As a rule, relatives wanted to take part in the Resource Group, and if they were accepted and validated in their role as a relative they could become a good resource for the client. The CM had a key role in creating a good emotional environment around the client, which is illustrated by the following quotation: “Above all, the visit to the mother meant a possibility for the client to listen to her mother's stories of years of frustration and to confirm that it can be difficult to be a relative.”

Relatives in this study were very important for the laying out and implementation of the crisis plan that was created for each client, a finding in keeping with previous experience (Falloon, Kydd, Coverdale, & Laidlaw, 1996). The early warning signs of a crisis were most often discovered first by a relative who could then sound the alarm to the CM and to someone in the health-care system so that measures could be taken that would prevent a psychotic event before it was too late. Relatives who earlier had not felt particularly involved in the community interventions with the client generally had a positive view of RACT, which gave them both greater influence on events centered on the client and more knowledge about contact, medication, functional hindrances, and psychosis.

### RACT program

The RACT program is built on an evidence-based foundation and has been adapted to Swedish context and conditions primarily by the Evidens Research and Development Center (Nordén et al., 2011). The clients' functional problems were recognized when they took on the task of working with the RACT material but, despite their problems, they worked very hard with both the interviews and the questionnaires. That they fulfilled their work assignments despite their limitations indicates that they were well motivated to take part in the program. Their difficulties are illustrated by the following quotation: “To carry out the patient interview took four meetings. Asking the client about the difficulties he had faced led to a long discussion concerning exactly what the word ‘difficulty’ means.” The students felt that the work book with modular descriptions was easy to follow. The purpose of putting the client in the center so that his or her self-sufficiency was strengthened did function as intended, to judge from the descriptions received, assuming that the modular assignments were taken on calmly and step by step by the clients. The material was also seen as being satisfactory given the training program's final goals, and the students discovered after completing the training that they had been able to make good use of the documentation for reconnecting and evaluation: “As one becomes more at home with the material so too does one discover the possibilities for going back to examine the common decisions made and see how they led to changes, both for the client and for the resource group.” Many related how they had followed the RACT materials step for step with their client, the family contact, and the resource group, which indicated that the client believed in the program. When working with the client began to take more time than expected or when difficulties arose, use was made of the advisor's resources and the activity in progress was modified in some way. An example is that the sequence in which modules were to be employed could be changed if the situation pointed to this need. A client might want to pursue a subject of great interest that was to have been taken up at a later time, but given the client's interest could be dealt with immediately in order to support that interest. It was also pointed out that use of the RACT materials had the result that participants viewed the control document more seriously in connection with work in progress. RACT appears to have functioned as intended, as Stefansson (2008) has stressed in his status report on the training efforts up to the date of his review.

Four tools were set forth as being of particular importance within RACT. The first tool concerns building alliances in order for shared efforts to reach the goals of the program: “Generally it may be said that the project is characterized by mutual respect and from my point of view also by a greater understanding of the client's difficulties and limitations.” The second tool is to offer specific elements of instruction for the client and the members of the Resource Group. Providing psycho-education about the illness and functional hindrances was essential in order for the treatment of the client to be as optimal as possible. The CM might, for example, have “informed the client's supervisors and colleagues, with the client's permission, of how a psychosis may appear.” A third tool is a model for solving problems, the so-called “six-step model,” which gives the client greater self-confidence and a greater sense of control. This model is applied in order to find solutions to reaching the partial goals that have been set by the client. The plans are formulated in the Resource Group, in which the collective competences surrounding the client are made use of: “A partial goal was to be able to empty the dishwasher. Together we worked out a solution.” Finally, crisis plans were designed in order to minimize the risk of a psychotic event, and these constituted an important tool. In the Resource Group a discussion was devoted to discussing which early warning signs were indicators of an imminent psychotic episode. It was important to try to clarify as concisely as possible what the observable behaviors were so that it would be possible to point them out directly to the client and family members and others close to the client. It was also important to modify the crisis plan when conditions changed: “The crisis plan was reworked three times during the course of the training program. One reason for reworking the crisis plan was the improvement in the client's condition and also that a vacation period was approaching.”

### The therapeutic circle

**Figure 1:**
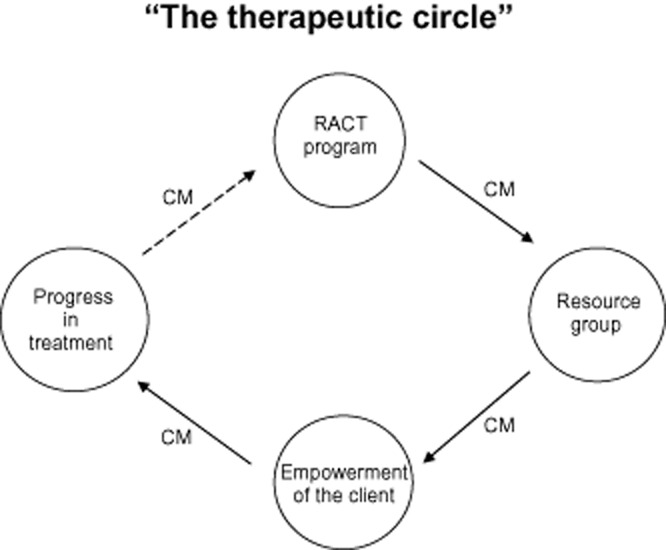
The five themes. The therapeutic circle starts off with the integrated care program Resource Group Assertive Community Treatment (RACT), which provides tools, methods, and structure that with the help of the case manager (CM) are conveyed to and used by the Resource Group. In a cooperative interaction with relatives, professionals, and the community and with the support of the CM, the client receives a greater self-awareness and experiences increased personal significance and quality of life. Through the empowerment of the client and repeated support contributions from the CM progre*ss* in treatment results. This is noted by the CM and provides the foundation for continued efforts following RACT.

One of the authors, who was not in any way involved in the retrieval of MUs or the formulation of the MUs categories, went through all of the synopses by index category in order to see if it was possible to identify recurring themes that together would describe the essence of the Case Managers' experiences during training in the integrated care program, RACT. Five such themes recurred in all seven index categories: (a) the RACT program; (b) the resource group; (c) the empowerment of the client; (d) progress in treatment; and (e) the case manager. The other three authors critically reviewed these recommendations and found that not only were the five themes to be found in all of the index categories but also that they were very well represented at the category and meaning levels, and that no other potential candidate for inclusion as a theme could match the five that had been recommended. The authors were therefore able to agree on the five themes and also found that the themes together created a therapeutic circle (see Figure 1) that begins with the RACT program, which contributes tools, methods and structure that, with the help of the CM, could be transmitted to the Resource Group and made use of there. As a result of interactions with relatives, professionals, and society, and the support of the CM, the client experienced a sense of increased self-awareness and a significant improvement in the quality of life. As a result of the client's active participation and strengthened empowerment, measurable improvements in treatment took place. These were registered by the CM and they created a foundation for continued efforts following the RACT. This result is in line with an earlier phenomenological study (Åsenlöf et al., 2007) in which a treatment method consisting of a combination of talk therapy, picture therapy and flotation-REST gave rise to a corresponding therapeutic circle for patients with stress-related complaints.

## Conclusion

It is clear that the Resource Group plays a central role in the RACT world as imagined by the Case Manager in training. It is there that the client first comes on stage as an actor and sets his or her goals, and it is there that empowerment of the client takes place. We distinguish the “larger Resource Group” from the “small resource group.” The greater or formal Resource Group consists of, in addition to the client, the case manager and, in the best case the physician, a number of people selected by the client as well. These may be family members, friends, acquaintances, neighbors, colleagues from the workplace, and professionals whom the client judges to be of special importance. In addition, a variety of professional experts such as psychologists, employment counselors, an advisor at the National Insurance Agency, residential support person or physical therapist might be temporarily connected to the group for a longer or shorter time. In this study, the Resource Group met five times during 1 year, on the average, which may be viewed as the typical frequency (Malm, 2002). The meeting usually lasted approximately 1 hr and only rarely more than 1.5 hr. A smaller part of the Resource Group, which in addition to the client and CM often consisted of persons close to the client, constitutes the “small resource group,” which usually met once per week. The Small Resource Group takes care of a number of ongoing tasks and there it is possible for the group to agree on a variety of training programs that the client needs, and from there it is common to contact people from the large Resource Group to help with these matters.

A limitation in the present study is that the research approach does not allow analyses of possible methodological artifacts due to the lack of control conditions. This must be elaborated in future studies. However, the RACT program has already been demonstrated to be an efficient and effective treatment strategy for people experiencing severe mental illness (e.g. Hayman-White & Happell, 2007; Nordén et al., 2011). In the ACT literature there is a lack of studies dedicated to qualitative methods and therefore it was considered interesting to conduct such a study with the purpose of investigating how trainee case managers experience a comprehensive educational curriculum.

Indeed, there are some limitations with a qualitative approach. An example of this is, as already noted, the absence of a control condition, and another example is the fact that it is not possible to make any generalizations from the results. Once this is said the major advantage of the qualitative method must also be recognized, that is the ability to observe and record phenomena that had been difficult to discover otherwise. It can relate to such observations that some of the components of the RACT material seem to have special significance. The circumstance that it is the client who defines his or her own treatment goals, nominates those to be included in the Resource Group and is trained by the case manager to be, if possible, the leader of the Resource Group, seem to be crucial factors in the empowerment of the client. The empowerment of the client, in turn, appears to be the major driving force for progress in treatment. Qualitative studies may function as generators of hypotheses, but it is of course necessary that those hypotheses are rigidly tested in subsequent future quantitative studies.

In the model, “the therapeutic circle,” which is singled out in the phenomenological analysis, it becomes clear that the CM has a key role in getting the different components of RACT to function together. A leading figure within the Swedish adaptation of RACT is the psychiatrist Ulf Malm, who has emphasized the necessity of providing qualitatively high level training, not only of those who are to serve as CMs within the RACT program, but also of the physician and the entire team. To work with the Resource-group's ACT may be compared with a sailing trip (Malm, 2002) on which the case manager is the helmsman. It is the client who determines the destination. The physician and the client's social network, all part of the Resource Group, constitutes the crew.
